# Reducing complexity: explaining inborn errors of metabolism and their treatment to children and adolescents

**DOI:** 10.1186/s13023-019-1236-9

**Published:** 2019-11-08

**Authors:** Nina A. Zeltner, Mendy M. Welsink-Karssies, Markus A. Landolt, Dominique Bosshard-Bullinger, Fabia Keller, Annet M. Bosch, Marike Groenendijk, Sarah C. Grünert, Daniela Karall, Beatrix Rettenbacher, Sabine Scholl-Bürgi, Matthias R. Baumgartner, Martina Huemer

**Affiliations:** 1 Division of Metabolism, University Children’s Hospital Zurich, Zurich, Switzerland; 2 Department of Psychosomatics and Psychiatry, University Children’s Hospital Zurich, Zurich, Switzerland; 3 Children’s Research Center, Zurich, Switzerland; 4 Department of Psychology, Division of Child and Adolescent Health Psychology, University of Zurich, Zurich, Switzerland; 5 Department of Pediatrics, Division of Metabolic Disorders, Emma Children’s Hospital, Amsterdam UMC – location AMC, Amsterdam, The Netherlands; 6 Stichting Stofwisselkracht, Haarlem, the Netherlands; 7 Department of General Pediatrics, Adolescent Medicine and Neonatology, Medical Center – University of Freiburg, Faculty of Medicine, Freiburg, Germany; 8 Clinic for Pediatrics, Medical University of Innsbruck, Innsbruck, Austria; 9 Weiberwirtschaft – Konzept und Gestaltung, Innsbruck, Austria; 10 Department of Paediatrics, Landeskrankenhaus Bregenz, Bregenz, Austria

**Keywords:** Disease knowledge, Patient education, Processing fluency, Self-efficacy, Coping, Disease management, Adolescence, Adherence

## Abstract

**Background:**

Inborn errors of metabolism (IEM) are a group of rare, heterogeneous and complex genetic conditions. Clinically, IEM often affect the central nervous system and other organs. Some carry the risk of progression and / or potentially life-threatening crises. Many patients have to adhere to lifelong dietary or drug treatment.

The complexity of IEM makes it difficult for patients and caregivers to understand their pathophysiology, inheritance and therapy rationale. Especially patients reaching adolescence may have only limited knowledge of their condition since medical care has often entirely been handled by their parents. Knowledge about disease and treatment, however, constitute pillars of self-responsible disease management. Not many standardized patient education materials on IEM are available and their comprehensibility has not been systematically investigated.

**Methods:**

We developed and tested patient education materials for school-aged children and adolescents with IEM. Informative texts and illustrations in paper form and as videos were developed by an international network of metabolic care professionals together with a graphic artist and experts for easy-to-read language. The materials were presented in standardized single or group training sessions to 111 individuals; first, to 74 healthy children and adolescents (recruited via public schools) and consecutively to 37 paediatric patients with IEM (phenylketonuria, galactosemia, urea cycle defects, lysosomal storage disorders) from six metabolic centres. Knowledge-gain was assessed by pre- and post-testing.

**Results:**

Knowledge-gain was significant in healthy children and adolescents as well as in patients (*p < .001*, *r* =. -77 /. -70). Effect sizes were large in both groups (*r* = -.77 / -.70). This result was independent from family language and teacher-rated concentration or cognitive capacity in healthy children.

**Conclusion:**

The newly developed patient education materials are a powerful tool to improve disease- and treatment-related knowledge. They facilitate communication between the medical team and children and adolescents with IEM and their caregivers.

## Background

Inborn errors of metabolism (IEM) are a group of rare, heterogeneous genetic conditions typically affecting enzyme functioning. Clinically, IEM often affect the central nervous system but also other organs. Some IEM carry the risk of “silent” progression while others cause phases of acute deterioration or even potentially life-threatening metabolic crises. Most patients carry a high burden of treatment as they require lifelong dietary and / or drug treatment (e.g. ammonia scavengers, substrate inhibitors, enzyme replacement). Adherence to lifelong dietary or medical treatment is demanding and requires joint efforts of patients and families, healthcare providers, and institutions such as kindergarten or school.

Recently, questionnaires specifically assessing health-related quality of life (HrQoL) of patients with phenylketonuria [1] and intoxication type disorders [2] have been developed. Interviews and focus groups with patients and parents are integral parts of the construction process of HrQoL instruments and allow insight into patients’ and parents’ perspectives. In focus groups and interviews conducted by our research group [3], patients and parents communicated that the complexity and abstractness of IEM and their treatments made it almost impossible for them to understand the pathophysiology or treatment rationale - let alone explain this to relatives, friends or teachers. Consequently, they reported feelings of frustration, isolation and helplessness in social situations.

Comprehension of disease, treatment mechanisms and outcome determinants is an important pillar of successful, self-responsible disease management [4]. Education materials for children and adolescents with IEM are sparse and their quality and comprehensibility have never been investigated.

Processing fluency - defined as the subjective ease with which an individual is able to process new external information - constitutes an important determinant of patient motivation, self-management and adherence [5, 6]. These factors are essential for optimal outcome and targets of patient education interventions [5, 7]. Standardized materials for continuous comprehensive, attractive, industry-independent education about the disease and its treatment are an unmet medical need for paediatric patients with IEM [8, 9]. It is well known that from age ten years, dietary adherence in phenylketonuria (PKU) and acute intoxication-type disorders (e.g. urea cycle disorders, maple syrup urine disease) declines [10]. This effect may, at least in part, be attributed to both the cumbersome transition of health responsibility from parents to patients during adolescence and limited knowledge about disease and treatment [11, 12].

This project aimed to develop and test specifically designed, standardized pictorial representations and videos combined with easy-to-understand texts for structured and comprehensible medical information on IEM for school-aged patients and their caregivers. We hypothesised that children and adolescents would be able to increase their knowledge about IEM after presentation of the materials.

## Methods

### Development of materials

Materials were developed in cooperation with a graphic artist (BR) and specialists for easy to read language (*Büro für Leichte Sprache, Lebenshilfe Bremen, Germany*). Six metabolic physicians, two psychologists and one patient representative were involved in the development process. They defined the contents necessary to understand the basics of IEM and were involved in several feedback loops to evaluate and refine the paper materials and videos.

### Materials

**Fig. 1:**
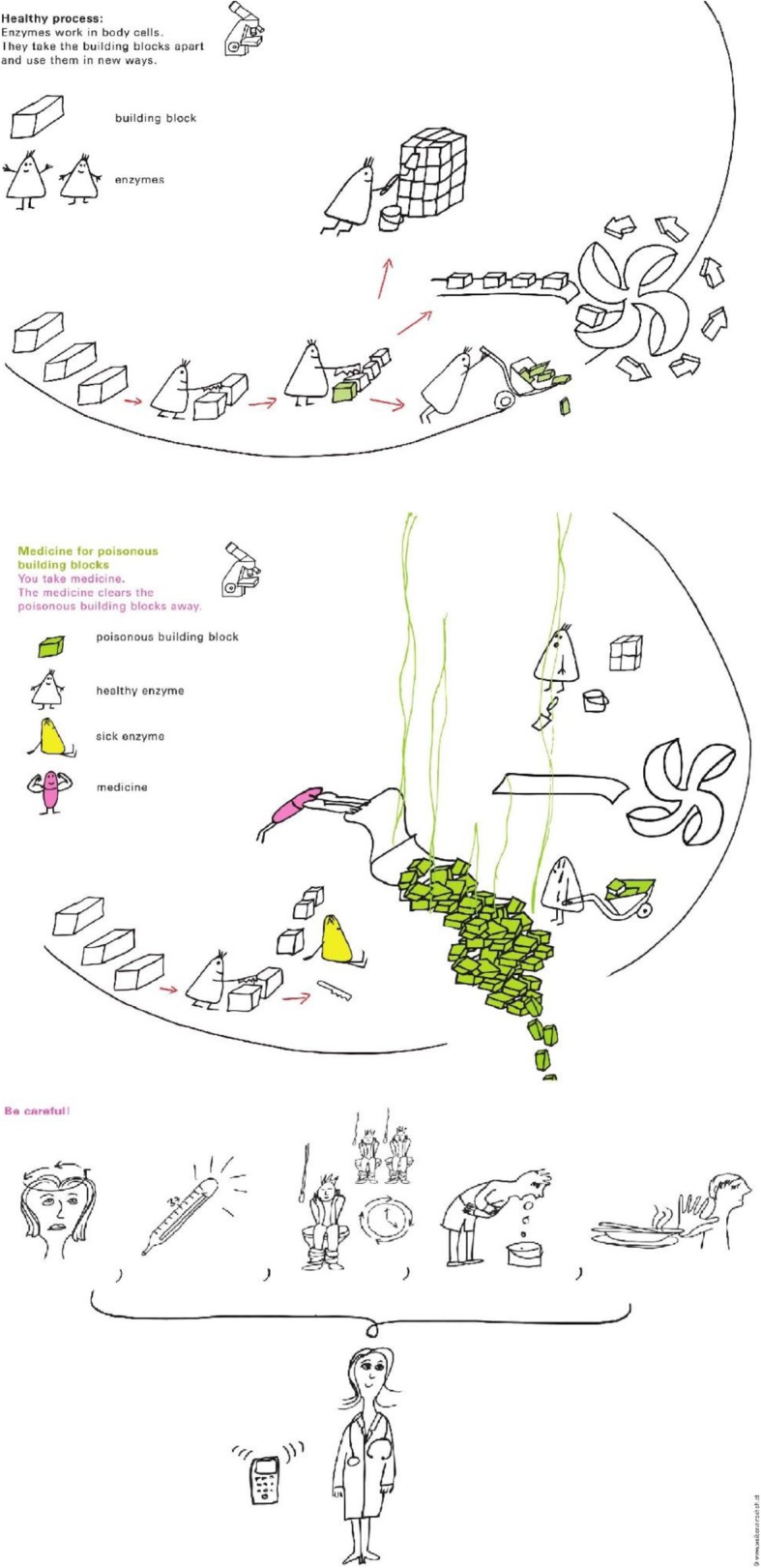
Example pictures (from top to bottom): (1) Enzymes at work in a healthy cell; (2) Medicine for intoxication-type disease; (3) Be careful! Recognise dangerous situations

Initially, a total of 23 modules were developed to explain different types of IEM, their inheritance and treatment. Each module consists of 1–2 pages of pictorial presentations with short easy-to-read text elements. The testing phase in healthy children and adolescents showed that the material explaining that the body is built of cells, which contain genetic information, were too complex. Hence, the content was reformatted and presented in two steps, leading to a final set of 24 modules for the second testing phase in patients. Contents of the modules are listed in Table 1. Figure 1 shows some examples (for more refer to [13]). Materials are available in English, Dutch, and German.

**Table 1:** Education modules explaining IEM and their treatment

Main domain	Content		Available as print version^a^	Available as video sequence^a^
The body	The body is built of cells		x	
	Cells contain the “body construction plan”		x	
Patterns of inheritance	Autosomal recessive		x	
	Autosomal dominant		x	
	X-chromosomal recessive		x	
	X-chromosomal dominant		x	
The healthy body	Building blocks for the body from foods and other sources		x	x
	Enzymes at work in a healthy cell		x	x
Enzyme defects	…in intoxication-type diseases		x	x
	…in storage diseases		x	x
	…in diseases where important substances cannot be produced		x	x
Treatment	Medicine	…for intoxication-type diseases	x	x
		…for storage diseases	x	x
		…for diseases where important substances cannot be produced	x	x
	Diet	…for intoxication-type diseases	x	x
		…for storage diseases	x	
		…for diseases where important substances cannot be produced	x	x
	Enzyme helper	…for intoxication-type diseases	x	x
		…for storage diseases	x	x
		…for diseases where important substances cannot be produced	x	
	Enzyme replacement	…for intoxication-type diseases	x	x
		…for storage diseases	x	x
		…for diseases where important substances cannot be produced	x	
Emergency situations	Be careful! Recognize dangerous situations (vomiting, fever etc.)		x	x

^a^Video sequences were produced based on the print versions, which were tested in healthy children and adolescents as well as patients. All materials are available at [13]

Modules were constructed as to be freely combined by the metabolic team to explain different inheritance patterns, IEM of different types (e.g. storage, intoxication), as well as a variety of treatment options (e.g. scavengers, enzyme replacement).

### Demographic data, abilities to concentrate and cognitive capacities

For all children and adolescents willing to participate, their parents completed a demographic questionnaire on basic information such as child age and family language. In the school setting we asked teachers to rate concentration and cognitive capacities. Ability to concentrate was rated on a four-point Likert-scale. Cognitive capacities were assessed by using three items with a six-point Likert-scale indicating fluid intelligence of the THINK 1–4 (Baudson, Wollschläger & Preckel, 2016; *Lehrpersonen-Einschätzungsskala zu den kognitiven Fähigkeiten; Test zur Erfassung der Intelligenz im Grundschulalter*). This scale was chosen for its shortness and good internal consistency of Cronbachs *α* = .95. Information about disease and school setting of the patients was part of the parent questionnaire.

### Comprehensiveness testing

To evaluate the materials, they were assembled for each testing session to sets of pictures explaining normal body functions, pattern of inheritance, enzyme function and dysfunction as well as treatment principles (for example normal enzyme functioning, autosomal recessive inheritance, intoxication of cells, dietary treatment and metabolite removal by drugs as a set for intoxication-type disorders). Disease-related knowledge was assessed before and after the teaching session using 5 to 8 specifically developed multiple choice items (number depending on content and age) and one open question. Test items of the knowledge test were developed by a metabolic physician, a psychologist, a psychology master student and a teacher. Piloting of the comprehensiveness test was performed in *n* = 41 children of 4 school classes, leading to a refinement of the test items and adjustment of complexity to avoid ceiling effects. Results of this pilot group are not included in the final analysis. After adaptation of the knowledge test, comprehensiveness testing was performed in healthy children and adolescents and later in patients. In both groups, materials were presented in a standardized teaching session given by trained persons with psychological and / or metabolic background.

#### Testing in healthy children and adolescents

Children and adolescents from 1st to 8th school grade (age 7 to 15 years) from 9 classes in 3 public schools in the Zurich area (Switzerland) were invited to participate. Teachers distributed information material and consent forms to all children attending their class for discussion with their parents. Inclusion criteria were informed consent by parents and child, sufficient command of German and absence of IEM in the family. Hence, this group is referred to as *the healthy group / healthy children and adolescents*. Two members of the study group with psychological background performed the testing (30-45 min per group). Modules were assembled according to different case vignettes which were presented to the groups. Consequently, all modules could be tested. Modules explaining inheritance were introduced only from the 5th grade upwards (> 11 years) due to their complexity and abstractness. Before and directly after the teaching session, the pre- and a post-knowledge-testing were performed.

#### Testing in patients

Convenience samples of patients with IEM from the metabolic clinics in Amsterdam (NL), Basel (CH), Bregenz (A), Freiburg (GE), Innsbruck (A) and Zurich (CH) were invited to participate. Patients were invited on the occasion of a scheduled visit at the outpatient clinic (all sites) and additionally during a patient group meeting (Amsterdam only). Testing was performed in individual patients or as a group testing with a maximum group size of 8 patients. Pre- and post-knowledge was tested before and directly after a teaching session with the materials. Duration of the procedure varied between 25 to 50 min. Inclusion criteria were sufficient command of the German (study sites in Austria, Germany, and Switzerland) or Dutch (study site in the Netherlands) language and the ability to participate in the test situation.

#### Statistical analyses

Data from the healthy group and the patient group were analysed independently, since materials were revised after the school-testing. Percent of correct answers were compared between pre- and post-testing to analyse knowledge gain in each of the two samples by Wilcoxon signed-rank tests. Answers to the open question counted double due to higher task complexity. Correlation coefficient *r* was calculated as an effect size measure for comparison of medians to define the magnitude of the difference between pre- and post-test results *(.10 small effect, .25 medium effect, .40 large effect)* [14, 15]. A significance level of *p* < 0.05 was defined to indicate statistical significance. Participants with more than ≥50% missing answers in one test were excluded from this analysis.

Spearman’s rank correlation was used to evaluate the relationship of knowledge gain and pre-test knowledge with potential influencing variables such as age, family language (same as language of the materials vs. other language), concentration and cognitive abilities (teacher’s rating; only available for the healthy group), diagnosis of galactosemia (subgroup of patient group),school type (regular school without additional assistance vs. schooling with assistance; patient group only) and testing site (country). Galactosemia diagnosis was chosen as variable in the patient group due to the well-established high frequency of impaired cognitive abilities associated with it [16].

All statistical analyses were performed with the statistical software package SPSS, versions 24.0 (IBM Corp. IBM SPSS Statistics for Windows).

### Subjective assessment of materials

Subjective assessment of materials (“how much do you like the materials?”) was asked on a three point smiley-scale at the end of the post-test questionnaire. Participants were encouraged to contribute further comments on the materials by an open question.

## Results

### Sample characteristics

One hundred and eleven children and adolescents were included in testing; 74 healthy children and adolescents (37 females; age range = 7.23–15.16; mean age = 11.56 ± 2.17) and 37 patients (19 females; age range = 6.96–19.18; mean age = 11.08 ± 3.17) with IEM (19 with phenylketonuria, 8 with galactosemia, 3 with propionic aciduria; 2 with hepatic glycogen storage diseases, 2 with Pompe disease, 1 each with mucopolysaccharidosis Type IVa, ornithin transcarbamylase deficiency and LCHAD). School setting (indicator for cognitive capacities) was regular school without additional assistance in 21 patients (57%) and schooling with assistance in 16 (43%) patients. One patient was excluded from the analyses due to ≥50% of missing answers in the post-test, but included for the subjective assessment of the materials.

### Testing of disease-related knowledge

**Fig. 2:**
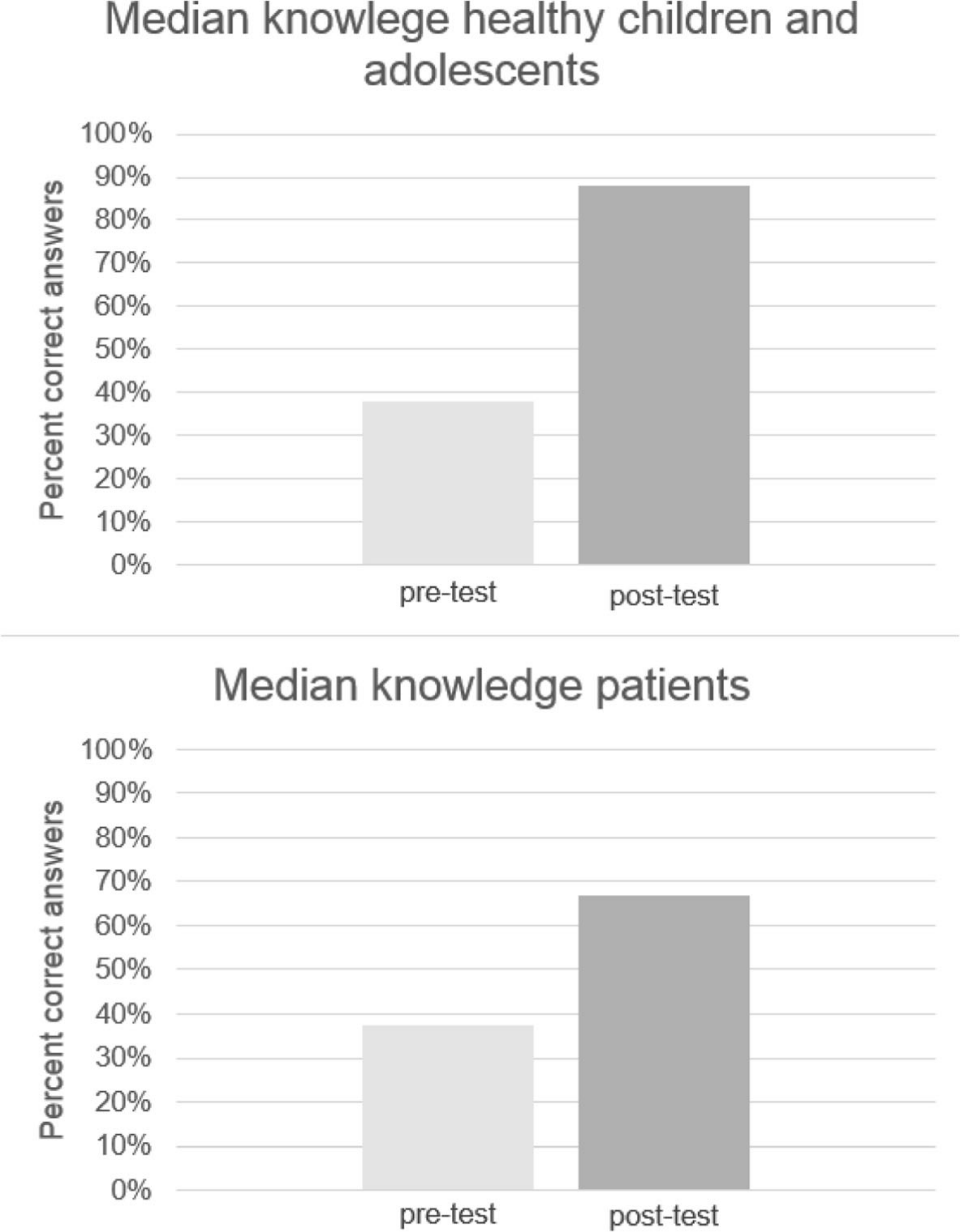
Knowledge gain in healthy children and adolescents and patients

Results of knowledge gain are depicted in Fig. 2. Healthy children and adolescents showed a significantly higher percentage of correct answers in the post-test (*Mdn* = 87.50) than in the pre-test (*Mdn* = 37.50; *z* = − 6.60, *p* = .000). This difference represents a large effect of *r =* − 0.77. Patients also had a higher percentage of correct answers in the post-test (*Mdn* = 66.67) compared to the pre-test (*Mdn* = 37.50; *z* = − 4.17, *p* = .000), which represents a large effect of *r* = −.70.

Correlations of knowledge gain with different variables are reported in detail in Table 2. In the healthy group, knowledge gain was not significantly related to age, family language, ability to concentrate or cognitive capacities. In patients, no significant relation was found between knowledge gain and family language, school type, and galactosemia diagnosis. A significant negative correlation was found between knowledge gain and age and knowledge gain and pre-test knowledge, while pre-test knowledge and age showed a significant positive correlation.

**Table 2:** Bivariate correlations of knowledge gain and pre-test knowledge with potential influencing variables

Healthy children and adolescents							
	1	2	3	4	5	6	
1. Knowledge gain	–	–	–	–	–	–	
2. Knowledge pre-test	− 0.77**	–	–	–	–	–	
3. Age	−0.16	0.46^**^	–	–	–	–	
4. Family language	− 0.19	0.11	0.18	–	–	–	
5. Cognitive capacities	0.10	0.06	−0.05	−0.19	–	–	
6. Ability to concentrate	0.15	0.00	−0.01	−0.09	0.71^**^	–	
Patients							
	1	2	3	4	5	6	7
1. Knowledge gain	–	–	–	–	–	–	–
2. Knowledge pre-test	−0.48^**^	–	–	–	–	–	–
3. Age	−0.53^**^	0.53^**^	–	–	–	–	–
4. Family language	0.04	−0.30	−0.12	–	–	–	–
5. Galactosemia	0.18	0.12	− 0.32	0.19	–	–	–
6. School type	−0.13	− 0.14	0.20	− 0.03	−0.27	–	–
7. Testing site	− 0.07	0.10	0.02	0.09	0.32	0.04	–

**Correlation is significant on the 0.01 level (2-tailed)

### Subjective assessment of materials

On a 3-point smiley scale, the majority of the healthy group (85%) and patients (84%) “liked” the materials, or rated them as “okay” (healthy group: 3%; patients 14%). None of the healthy children and adolescents and only one patient disliked the materials (3%). Nine healthy children and adolescents (12%) did not answer this question.

## Discussion

Use of unexplained medical jargon with families and patients is still common in the paediatric field [17] although it has been shown that information, which can be processed fluently, is considered more trustworthy, honest and safe and that the recipient is more inclined and likely to fulfil inferred tasks [5]. Processing fluency depends mainly on perceptive fluency, determined by visual display and linguistic fluency, which depends on wording and grammatical complexity. Our materials were designed to break down complicated concepts by pictorial representations and easy-to-read language texts to achieve good processing fluency for this complex information [5].

A main strength of our newly developed modular educational materials is that they have been thoroughly tested not only in patients from different countries, but also in naïve healthy children and adolescents. The materials can be used to educate IEM patients from school age and parents with any educational background and family language on different types of IEM (e.g. storage or intoxication type). They follow a modular system and thus allow for flexible combinations adaptable to individual cases, age groups and intellectual capacities, in a step-by-step approach.

Standardized teaching with our newly developed pictorial materials had a positive influence on disease-related knowledge in healthy children and in patients indicating good retrieval fluency of the materials. Knowledge gain was not related to age, family language and teacher’s ratings of concentration abilities and cognitive capacities in the healthy group. Similarly, family language and indicators for cognitive capacities (galactosemia, school type) had no influence on knowledge gain in the patient group. However, knowledge gain was negatively related to age and pre-test-knowledge in patients. These results argue against our hypothesis that adolescents have little disease knowledge.

Experience during the testing sessions in healthy children showed that the rather abstract information e.g. about the body consisting of cells and genetics (DNA, genes, inheritance) were difficult to convey to younger children but worked well for adolescents. The contents how enzymes work and what happens when an enzyme is not working properly as well as treatment principles (e.g. diet, enzyme replacement) and information on how to recognise and act on emergencies were easier to grasp. Thus, materials about enzyme function, treatment options and how to act in emergency situations can be introduced in children from about 7 years of age while the more abstract topics (e.g. inheritance patterns) can be discussed in adolescence and / or with parents.

Although we controlled for potential effects of cognitive abilities on the understanding of the materials by different variables, a limitation of our study is that only information with regard to the attendance of special education was collected for the included patients. Therefore, we were not able to assess the effect of (severe) cognitive deficits on the test results. The short video sequences that were developed based on the cartoons may be helpful for teaching both younger patients and patients with limited cognitive abilities, but this was not specifically tested. Furthermore, our teaching settings with groups of patients may not be representative for the usual clinical setting. We hypothesize, however, that the results should be replicable or may even be better in a one-to-one setting, which allows to fully focus on the needs of an individual / a family.

It is known that in patients with chronic diseases effective health education does not only improve knowledge, but also supports adherence to treatment and successful self-management, by influencing the patients’ attitude and daily practice [17, 18]. All these factors are essential for optimal outcome in patients with IEM [5, 7]. In this study, so far, we only tested short-term knowledge gain. The effect of our materials on adherence, daily practice and biochemical outcome markers will be investigated in a next step. To allow wider use of the materials they will be accessible for professionals, patients and their families on an attractive patient-oriented homepage, which is another focus of this ongoing project.

Medical jargon and incomprehensive disease information constitute a barrier to informed and shared decision-making [17]. Over 80% of the study participants indicated that they liked the materials and appreciated them as an indicator of a respectful approach of care. Facilitated communication between patient and metabolic care team may not only enhance patients’ safety, outcome, attitude towards the care team and quality of life but does significantly reduce the workload of metabolic professionals due to increased independence of patients and families [5]. Involvement of parents in teaching sessions and provision of the materials enables parents to explain the contents repeatedly to their child at home and to involve siblings, other family members or external caregivers to gain social support.

## Conclusions

The newly developed materials are a powerful tool to enhance disease-related knowledge and processing fluency in children and adolescents with IEM from school age, preparing the ground for participative, safe and easy communication between patients and the metabolic team.

## Data Availability

All data generated or analysed during this study are included in this published article. More information about the materials is available at www.kispi.uzh.ch/fzk/stoffwechselpsychologie . Physicians or researchers interested in using the materials may contact the corresponding author.
